# Contemporary Trends in Axillary Surgery for ER-Positive, HER2-Negative Breast Cancer Stratified by Neoadjuvant Endocrine Therapy, Neoadjuvant Chemotherapy, or Upfront Surgery

**DOI:** 10.1245/s10434-025-18225-5

**Published:** 2025-09-23

**Authors:** Sasha R. Douglas, Samantha M. Thomas, Akiko Chiba, Maggie L. DiNome, Gayle A. DiLalla, E. Shelley Hwang, Kendra Modell Parrish, Laura H. Rosenberger, Jennifer K. Plichta, Ton Wang, Astrid M. Botty van den Bruele

**Affiliations:** 1 Department of Surgery, Duke University, Durham, NC USA; 2 Biostatistics and Bioinformatics, Duke University, Durham, NC USA

## Abstract

**Background:**

De-escalation of axillary surgery for hormone receptor-positive breast cancer has gained traction, but guidelines for axillary management after neoadjuvant endocrine therapy (NET) remain ill-defined.

**Methods:**

Female patients age ≥50 years with clinical T1-4c, N0-1, ER+/HER2–<PUB4> breast cancer (2012–2021) were selected from the National Cancer Database (NCDB) and divided into three groups based on treatment: upfront surgery, neoadjuvant chemotherapy (NAC), or NET. Axillary surgery was categorized as no axillary surgery, sentinel lymph node biopsy (SLNB) alone, sentinel lymph node biopsy followed by axillary lymph node dissection (ALND), or ALND alone. Descriptive analysis and logistic regression were used to identify factors associated with ALND, and survival analysis was performed.

**Results:**

The inclusion criteria were met by 792,581 patients. The majority underwent surgery first (94.3 %), whereas 2.6 % received NAC and 3.1 % received NET. After adjustment, the odds of undergoing ALND were shown to be higher for those receiving NAC (odds ratio [OR], 1.18; 95 % confidence interval [CI], 1.12–1.24) or NET (OR, 1.10; 95 % CI, 1.05–1.14) than for those receiving surgery first (*p* < 0.001). The patients receiving NET were less likely to convert from cN+ to ypN0 (8.3 % vs NAC 19.6 %; *p* < 0.001), but they still were less likely to undergo ALND after SLNB. Overall survival was highest among the patients in the surgery-first group regardless of clinical nodal status (*p* < 0.001).

**Conclusions:**

Although the patients receiving NET had lower rates of nodal pathologic complete response, this did not translate to higher conversion to ALND after SLNB, suggesting that providers do not interpret residual nodal disease after NET the same way as after NAC.

**Supplementary Information:**

The online version contains supplementary material available at 10.1245/s10434-025-18225-5.

De-escalation of axillary surgery has been the trend in the contemporary management of breast cancer. Axillary lymph node dissection (ALND) has been increasingly replaced with sentinel lymph node biopsy (SLNB) or omission of axillary staging entirely.^1–3^ High-quality evidence exists to demonstrate that for women with clinically negative nodes, the use of SLNB rather than ALND does not have a negative impact on oncologic outcomes.^4–7^ Additionally, for those with clinically negative nodes, low-volume axillary nodal positivity after upfront surgery does not require a completion ALND.^6,8^ Even for patients with clinically positive nodes who convert to clinically negative nodes after neoadjuvant chemotherapy (NAC), the ALND rates are declining since the results from three large prospective trials demonstrated the feasibility of SLNB for select patients who convert to clinical node negativity after NAC.^9–11^

Hormone receptor-positive (HR+) breast cancer is the most common subtype, with about 80 % of breast cancers classified as estrogen receptor-positive (ER+) and approximately 65 % also classified as progesterone receptor-positive (PR+).^12^ For this subtype, neoadjuvant endocrine therapy (NET) can be used to treat patients with locoregionally advanced breast cancers who may be poor surgical candidates or to convert previously breast-conserving surgery (BCS)-ineligible patients to BCS eligibility.^13^ Evidence shows that in these cases, NET has similar efficacy with much less toxicity compared with neoadjuvant chemotherapy (NAC).^14^

However, HR+ (defined as ER+ and/or PR+)/human epidermal growth factor receptor 2-negative (HER2–) tumors have lower rates of nodal pathologic complete response (pCR) after NAC than triple-negative or HER2+ disease.^15^ The ACOSOG Z1071 trial reported nodal PCR rates for biopsy-proven node-positive HR+/HER2– breast cancer after NAC to be about 21 %, but NET was not included in this analysis.^16^ Consequently, it is unclear whether the current guidelines for treatment of the axilla after NAC should be applied to patients receiving NET, and very little literature has addressed this question to date **(**Table 1).^17–20^ Specifically, the role of ALND for patients with residual nodal disease after NET warrants clarification.

**Table 1:** Studies discussing axillary management for breast cancer after NET

Study	No. of patients	Type of study	Patient population studied	Key points
Weiss et al.^17^ (2019)	92,204	Retrospective observational cohort	Stage II or III HR+/HER2– breast cancer from the NCDB (2012–2015)	Patients with cN0 or cN1 disease treated with NET were more likely to have residual nodal disease, but less likely to undergo cALND than those treated with NAC Outcomes data are needed to guide optimal axillary management for this patient population.
Stafford et al.^18^ (2020)	1479 (systematic review) 4580 (NCDB)	Mixed methods: systematic review, retrospective observational cohort study	Node-positive HR+/HER2– breast cancer patients receiving NET >30 days before surgery in the NCDB (2010–2016)	14.5 % of NET patients achieved pCR Recommend NET be considered for axillary downstaging
Kantor et al.^20^ (2021)	94 (DFBWCC) 4363 (NCDB)	Retrospective observational cohort (2 datasets)	cT1-4N0-1M0 HR+/HER2– breast cancer from Dana-Farber/Brigham and Women's Cancer Center cohort (DFBWCC) (2015–2018) and NCDB cohort (2012–2016)	>90 % cN0 patients receiving had fewer than three positive nodes at surgery Overall survival was equal for SLNB vs ALND for any amount of residual nodal disease Recommend cN0 NET patients be treated like surgery-first patients
Murphy et al.^19^ (2021)	186	Retrospective observational cohort	ER+ breast cancer treated with NET for a minimum of 30 days before surgery between October 2008 and November 2019 from Mayo Clinic prospective breast surgery registry	Of cN+ ypN+ patients, 16.7 % were managed without ALND; no nodal recurrences after median follow-up of 35 months. Recommend NET patients with low-volume axillary disease be treated like surgery first-patients

NET, neoadjuvant endocrine therapy; HR, hormone receptor; HER2, human epidermal growth factor receptor 2; cALND, completion axillary lymph node dissection; NCDB, National Cancer Database; pCR, pathologic complete response; DFBWCC; SLNB, sentinel lymph node biopsy; ER, estrogen receptor

To this end, we used the National Cancer Database (NCDB) to assess the state of axillary management trends for patients with HR+ breast cancer undergoing upfront surgery or receiving NAC or NET. Specifically, we estimated the rates of no axillary surgery, SLNB, and ALND by systemic therapy type with attention to how patient demographics and tumor characteristics may be associated with these rates.

## Methods

**Fig. 1:**
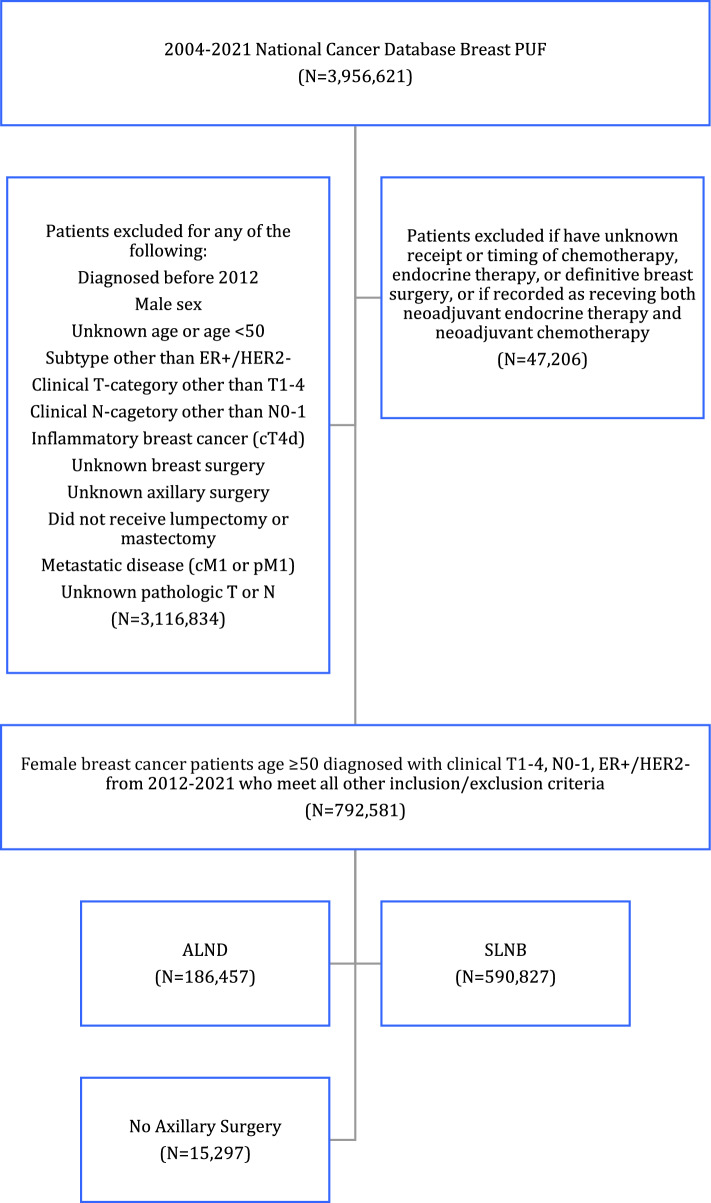
Patient selection diagram.

Female patients age ≥50 years with a diagnosis of clinical T1-4c, N0-1, ER+/HER2– breast cancer from 2012 to 2021 were selected from the NCDB (fall 2023 data release). The study excluded patients with inflammatory breast cancer (clinical T4d) or metastatic disease (clinical M1 or pathologic M1), as well as those who did not undergo lumpectomy or mastectomy. Patients with missing or unknown breast surgery, axillary surgery, pathologic T or N category, time from diagnosis to definitive breast surgery, chemotherapy receipt and timing, or endocrine therapy receipt and timing also were excluded (Fig. 1).

The study defined NET as receipt of endocrine therapy before definitive breast surgery and NAC as receipt of chemotherapy before definitive surgery. Patients recorded as receiving both NET and NAC were excluded. Time to treatment was defined as the time from diagnosis to chemotherapy for patients who received NAC, as time fom diagnosis to endocrine therapy for patients who received NET, and as time from diagnosis to definitive breast surgery for those who had surgery first. Axillary surgery was defined as described in the NCDB data dictionary, variable *RX_SUMM_SCOPE_REG_LN_2012,* as follows: code 0 (none), code 2 (SLNB alone), codes 3 to 5 (ALND alone), and codes 6 and 7 (SLNB + ALND).

The axillary surgery group was designated as none, SLNB, or ALND (ALND alone or SLNB + ALND). Targeted axillary dissection is not coded separately in the NCDB and likely would be classified under SLNB unless a completion lymph node dissection was subsequently performed, in which case it likely would be classified as SLNB + ALND.

Patient characteristics were summarized as number (%) for categorical variables and as median (interquartile range [IQR]) for continuous variables. Differences were tested with chi-square for categorical variables and with analysis of variance (ANOVA) for continuous variables. Logistic regression was used to identify factors associated with undergoing ALND versus not undergoing ALND (SLNB alone or none).

This model was built in the generalized estimating equations framework and included an exchangeable covariance structure to account for the correlation of patients treated at the same facility. The following covariates were included in the logistic model: age, Charlson-Deyo comorbidity score, clinical T category, clinical N category, facility type, facility location, insurance type, race and ethnicity, year of diagnosis, LVI, and treatment group.

Odds ratios (ORs) and 95 % confidence intervals (CIs) are reported. Unadjusted overall survival was estimated with the Kaplan-Meier method, and log-rank tests were used to compare survival between groups. Follow-up time was estimated with the reverse Kaplan-Meier method.

No adjustments were made for multiple comparisons. Only patients with complete data were included in the logistic regression model, and the model sample size is reported. All statistical analyses were performed with SAS version 9.4 (SAS Institute, Cary, NC, USA). This project was deemed exempt from institutional review board (IRB) review because it used only de-identified, publicly available data from the NCDB in accordance with institutional policies.

## Results

The study identified 792,581 female patients age ≥50 years with a diagnosis of clinical T1-4c, N0-1, ER+/HER2– breast cancer from 2012 to 2021 who met all the other inclusion and exclusion criteria (Fig. 1). Of these patients, 94.3 % (747,659) underwent surgery first, 2.6 % (20,704) received NAC, and 3.1 % (24,218) received NET (Table 2). Regarding axillary surgery, 74.5 % (*n* = 590,827) underwent SLNB, 23.5 % (*n* = 186,457) underwent ALND, and 1.9 % (*n* = 15,297) had no axillary surgery. The overall rate of ALND decreased from 35.0 % in 2012 to 13.5 % in 2021. Stratification by clinical nodal status showed that ALND use declined from 30.3 to 10.6 % among the patients with cN0 disease and from 84.7 % to 69.6 % among the patients with cN1 disease. The rate of ALND at academic centers was 23.2 % versus 27.8 % at community centers. The rate of ALND was 78.5 % for cN1 disease compared with 19.6 % for cN0 disease and 44.5 % if LVI was present compared with 19.5 % if LVI was absent (*p* < 0.001).

**Table 2:** Patient, tumor, and treatment characteristics by treatment group using the NCDB 2012–2021

	All patients (*n* = 792,581) *n* (%)^a^	Treatment Group			*p* value
		NAC (*n* = 20,704) *n* (%)^a^	NET (*n* = 24,218) *n* (%)^a^	Surgery first (*n* = 747,659) *n* (%)^a^	
*Age (years)*					<0.001
50–69	513,400 (64.8)	18,122 (87.5)	14,721 (60.8)	480,557 (64.3)	
70+	279,181 (35.2)	2582 (12.5)	9497 (39.2)	267,102 (35.7)	
Median (IQR)	66 (59–72)	59 (54–65)	67 (60–74)	66 (59–73)	<0.001
*Race and ethnicity*					<0.001
Hispanic	37,054 (4.7)	1401 (6.8)	1318 (5.4)	34,335 (4.6)	
Non-Hispanic Asian	23,516 (3)	669 (3.2)	807 (3.3)	22,040 (2.9)	
Non-Hispanic black	64,809 (8.2)	2815 (13.6)	2317 (9.6)	59,677 (8)	
Non-Hispanic white	641,167 (80.9)	15,122 (73)	18,990 (78.4)	607,055 (81.2)	
Other	7585 (1)	232 (1.1)	258 (1.1)	7095 (0.9)	
Unknown^b^	18,450 (2.3)	465 (2.2)	528 (2.2)	17,457 (2.3)	
*Charlson-Deyo comorbidity score*					<0.001
0	635,991 (80.2)	17,447 (84.3)	18,995 (78.4)	599,549 (80.2)	
1	113,421 (14.3)	2551 (12.3)	3521 (14.5)	107,349 (14.4)	
2+	43,169 (5.4)	706 (3.4)	1702 (7)	40,761 (5.5)	
*Year of diagnosis*					<0.001
2012–2016	378,016 (47.7)	15,237 (73.6)	9851 (40.7)	352,928 (47.2	
2017–2021	414,565 (52.3)	5467 (26.4)	14,367 (59.3)	394,731 (52.8)	
*Facility type*					<0.001
Academic/research	224,541 (28.3)	6639 (32.1)	7970 (32.9)	209,932 (28.1)	
Community	58,329 (7.4)	1248 (6)	1421 (5.9)	55,660 (7.4)	
Comprehensive community	334,681 (42.2)	8054 (38.9)	9080 (37.5)	317,547 (42.5)	
Integrated network	175,030 (22.1)	4763 (23)	5747 (23.7)	164,520 (22)	
*Histology*					<0.001
Ductal	581,668 (73.4)	15,585 (75.3)	16,259 (67.1)	549,824 (73.5)	
Lobular	177,761 (22.4)	4565 (22)	7036 (29.1)	166,160 (22.2)	
Other	33,152 (4.2)	554 (2.7)	923 (3.8)	31,675 (4.2)	
*Clinical T category*					<0.001
T1	611,721 (77.2)	4841 (23.4)	13,805 (57)	593,075 (79.3)	
T2	157,584 (19.9)	10,013 (48.4)	7938 (32.8)	139,633 (18.7)	
T3	18,436 (2.3)	4075 (19.7)	1754 (7.2)	12,607 (1.7)	
T4	4840 (0.6)	1775 (8.6)	721 (3)	2344 (0.3)	
*Clinical N category*					<0.001
N0	739,621 (93.3)	8890 (42.9)	20,674 (85.4)	710,057 (95)	
N1	52,960 (6.7)	11,814 (57.1)	3544 (14.6)	37,602 (5)	
*Pathologic T category*					<0.001
T0	2652 (0.3)	1590 (7.7)	126 (0.5)	936 (0.1)	
T1	576,208 (72.7)	8804 (42.5)	14,328 (59.2)	553,076 (74)	
T1IS	1416 (0.2)	373 (1.8)	87 (0.4)	956 (0.1)	
T2	183,995 (23.2)	7127 (34.4)	7568 (31.2)	169,300 (22.6)	
T3	24,203 (3.1)	2229 (10.8)	1720 (7.1)	20,254 (2.7)	
T4	4107 (0.5)	581 (2.8)	389 (1.6)	3137 (0.4)	
*Pathologic N category*					<0.001
N0	622,061 (78.5)	8271 (39.9)	16,506 (68.2)	597,284 (79.9)	
N1	107,901 (13.6)	7187 (34.7)	4723 (19.5)	95,991 (12.8)	
N1Mic	30,595 (3.9)	889 (4.3)	1057 (4.4)	28,649 (3.8)	
N2	22,403 (2.8)	3121 (15.1)	1323 (5.5)	17,959 (2.4)	
N3	9621 (1.2)	1236 (6)	609 (2.5)	7776 (1)	
Median tumor size: cm (IQR)	1.4 (0.9–2.2)	3 (2–4.9)	1.8 (1.1–3)	1.4 (0.9–2.1)	<0.001
Median no. of LNs retrieved (IQR)	2 (1–4)	7 (3–13)	3 (2–5)	2 (1–4)	<0.001
Median no. of positive LNs (IQR)	0 (0–0)	1 (0–3)	0 (0– 1)	0 (0–0)	<0.001
*Grade*					<0.001
1	247,278 (31.2)	2050 (9.9)	7303 (30.2)	237,925 (31.8)	
2	411,649 (51.9)	9878 (47.7)	13,583 (56.1)	388,188 (51.9)	
3	113,378 (14.3)	7572 (36.6)	2695 (11.1)	103,111 (13.8)	
Unknown^b^	20,276 (2.6)	1204 (5.8)	637 (2.6)	18,435 (2.5)	
*Lymphovascular invasion*					<0.001
Absent	603,664 (76.2)	10,618 (51.3)	17,436 (72)	575,610 (77)	
Present	102,909 (13)	5731 (27.7)	3402 (14)	93,776 (12.5)	
Unknown^b^	86,008 (10.9)	4355 (21)	3380 (14)	78,273 (10.5)	
*PR status*					<0.001
PR+	703,692 (88.8)	16,089 (77.7)	21,498 (88.8)	666,105 (89.1)	
PR–	88,330 (11.1)	4602 (22.2)	2708 (11.2)	81,020 (10.8)	
Unknown^b^	559 (0.1)	13 (0.1)	12 (0)	534 (0.1)	
*Axillary surgery*					<0.001
ALND	186,457 (23.5)	13,053 (63)	7183 (29.7)	166,221 (22.2)	
SLNB alone	590,827 (74.5)	7418 (35.8)	16,325 (67.4)	567,084 (75.8)	
None	15,297 (1.9)	233 (1.1)	710 (2.9)	14,354 (1.9)	
*Chemotherapy type*					<0.001
None	644,780 (81.4)	0 (0)	20,972 (86.6)	623,808 (83.4)	
Adjuvant	127,097 (16)	0 (0)	3246 (13.4)	123,851 (16.6)	
Neoadjuvant	20,704 (2.6)	20,704 (100)	0 (0)	0 (0)	
*Endocrine therapy type*					<0.001
None	97,206 (12.3)	2265 (10.9)	0 (0)	94,941 (12.7)	
Adjuvant	671,157 (84.7)	18,439 (89.1)	0 (0)	652,718 (87.3)	
Neoadjuvant	24,218 (3.1)	0 (0)	24,218 (100)	0 (0)	
*Treatment with radiation*					<0.001
No	279,906 (35.3)	4966 (24)	10,808 (44.6)	264,132 (35.3)	
Yes	485,221 (61.2)	14,779 (71.4)	12,507 (51.6)	457,935 (61.2)	
Unknown^b^	27,454 (3.5)	959 (4.6)	903 (3.7)	25,592 (3.4)	
*Surgery type*					<0.001
Lumpectomy	549,896 (69.4)	7630 (36.9)	13,595 (56.1)	528,671 (70.7)	
Mastectomy	242,685 (30.6)	13,074 (63.1)	10,623 (43.9)	218,988 (29.3)	
Median time from diagnosis to treatment: months (IQR)	1.18 (0.79–1.71)	1.18 (0.82–1.68)	0.95 (0.53–1.68)	1.18 (0.79–1.71)	<0.001
Median time from start of endocrine therapy to definitive surgery: months (IQR)^c^	1.81 (0.95–4.14)		1.81 (0.95–4.14)		-
Median follow-up: months (95 % CI)	62.5 (62.4–62.6)	72.4 (71.9–72.9)	53.1 (52.5–53.8)	62.4 (62.3–62.6)	<0.001

NCDB, National Cancer Database; NAC, neoadjuvant chemotherapy; NET, neoadjuvant endocrine therapy; IQR, interquartile range; LN, lymph node; PR, progesterone receptor ALND, axillary lymph node dissection; SLNB, sentinel lymph node biopsy; CI, confidence interval

^a^Percentages may not add up to 100 due to rounding or missing values

^b^Unknown values are not included in *p* value estimation

^c^Among NET patients only

The baseline median tumor size for the patients undergoing ALND was 1.9 cm (IQR, 1.2–3.0 cm) versus 1.3 cm (IQR, 0.9–2.0 cm) for the patients undergoing SLNB and 1.2 cm (IQR, 0.8–2.0 cm) for the patients not undergoing axillary surgery (Table S1). The median time from diagnosis to treatment was shorter in the NET group (0.95 months; IQR, 0.53–1.68 months) than in the NAC group (1.18 months; IQR, 0.82–1.68 months) or the surgery-first group (1.18 months;, IQR, 0.79–1.71 months) (*p* < 0.001) The patients who received NAC were more likely to receive adjuvant radiation (71.4 %) than surgery first (61.2 %) or NET (51.6 %) (*p* < 0.001; Table 2).

**Fig. 2:**
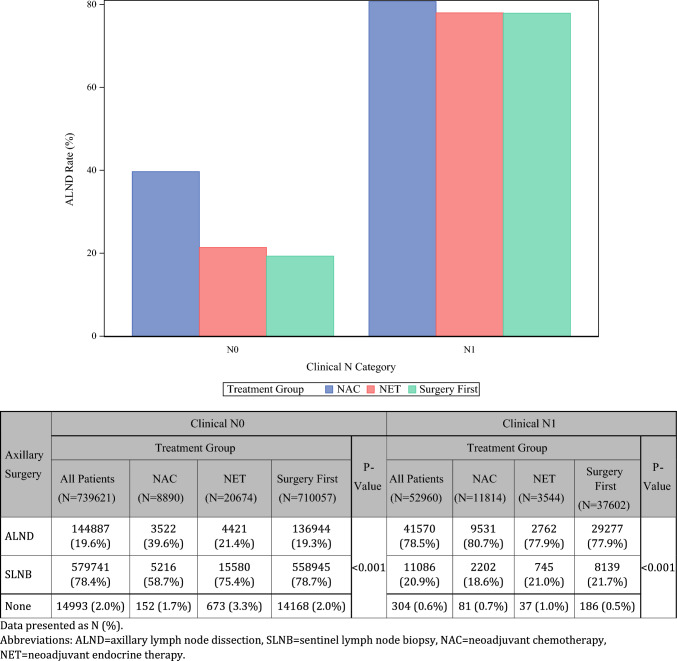
Axillary surgery rates by treatment group (NAC, NET, or surgery first) and clinical N category

Of the patients who received NAC, 63.0 % underwent ALND compared with 29.7 % of those receiving NET and 22.2 % of those undergoing surgery first (*p* < 0.001; Table 2). The patients receiving NAC had the highest rates of ALND in both cN0 and cN1 disease (39.6 % and 80.7 %, respectively). For those with clinically node-positive disease at presentation, the rates of ALND after NET did not differ from that for surgery first (77.9 % for both; Fig. 2).

In addition to treatment group, several other factors were significantly associated with receipt of ALND. Larger tumor size (T2-4) and clinically node-positive disease were strongly associated with higher odds of receiving ALND. The presence of LVI also significantly increased the likelihood of ALND (OR, 2.08; 95 % CI, 2.03–2.13; *p* < 0.001). Younger age, black race, and higher comorbidity scores were associated with increased ALND rates. Conversely, treatment at an integrated network or at institutions in the West or Northeast were associated with lower odds of ALND. After adjustment for such variables affecting ALND rates, the odds of undergoing ALND were higher for those receiving NAC (OR, 1.18; 95 % CI, 1.12–1.24) and, to a lesser degree, for those receiving NET (OR, 1.10; 95 % CI, 1.05–1.14) compared with those receiving surgery first (*p* < 0.001; Table 3).

**Table 3:** Adjusted logistic regression for ALND vs SLNB or no axillary surgery (*n* = 684,890)

	OR (95 % CI)	*p* value	Overall *p* value
*Age (years)*			<0.001
50–69	Reference		
70+	0.95 (0.936–0.963)	<0.001	
*Race and ethnicity*			<0.001
Non-Hispanic white	Reference		
Hispanic	1.043 (1.009–1.078)	0.01	
Non-Hispanic Asian	0.975 (0.942–1.01)	0.16	
Non-Hispanic black	1.098 (1.074–1.123)	<0.001	
Other	1.051 (0.991–1.114)	0.10	
*Insurance type*			0.04
Private	Reference		
Government	0.99 (0.976–1.004)	0.15	
None	1.067 (1.003–1.136)	0.04	
*Charlson/Deyo comorbidity score*			<0.001
0	Reference		
1	1.052 (1.033–1.071)	<0.001	
2+	1.075 (1.046–1.105)	<0.001	
Year of diagnosis	0.871 (0.865–0.877)	<0.001	<0.001
*Facility type*			<0.001
Academic/research	Reference		
Community	1.121 (0.956–1.314)	0.16	
Comprehensive community	0.991 (0.85–1.156)	0.91	
Integrated network	0.837 (0.717–0.976)	0.02	
*Facility location*			0.001
South	Reference		
Midwest	0.94 (0.832–1.062)	0.32	
Northeast	0.841 (0.729–0.97)	0.02	
West	0.754 (0.654–0.869)	<0.001	
*Clinical T category*			<0.001
T1	Reference		
T2	1.616 (1.589–1.644)	<0.001	
T3	3.003 (2.869–3.144)	<0.001	
T4	2.938 (2.676–3.225)	<0.001	
*Clinical N category*			<0.001
N0	Reference		
N1	9.364 (8.851–9.905)	<0.001	
*Treatment group*			<0.001
Surgery first	Reference		
NAC	1.177 (1.117–1.24)	<0.001	
NET	1.095 (1.054–1.137)	<0.001	
*Lymphovascular invasion*			<0.001
Absent	Reference		
Present	2.08 (2.031–2.13)	<0.001	

ALND, axillary lymph node dissection; SLNB, sentinel lymph node biopsy; OR, odds ratio; CI, confidence interval; NAC, neoadjuvant chemotherapy; NET, neoadjuvant endocrine therapy

Among the clinically node-positive patients, 19.6 % were found to be pathologically node-negative after receiving NAC, whereas this was true for only 8.3 % of those receiving NET (*p* < 0.001). An ALND alone was performed for 55.4 % of the surgery-first group, 54.9 % of the NAC group, and 56.4 % of the NET group. Interestingly, of the clinically node-positive patients who underwent SLNB and were found to have residual nodal disease, 70.5 % in the NAC group went on to completion ALND compared with 57.5 % in the NET group (*p* < 0.001; Table 4).

**Table 4:** Pathologic nodal status and axillary surgery of cN+ patients

	NAC *n* (%)^a^	NET *n* (%)^a^	Surgery first *n* (%)^a^	*p* value
*Pathologic nodal status*				<0.001
N0	2314 (19.6)	295 (8.3)	2415 (6.4)	
N+	9500 (80.4)	3249 (91.7)	35,187 (93.6)	
*Axillary surgery for pN+ patients*				<0.001
None	65 (0.7)	29 (0.9)	147 (0.4)	
SLNB alone	1244 (13.1)	590 (18.2)	6604 (18.8)	
ALND alone	5218 (54.9)	1831 (56.4)	19,501 (55.4)	
SLNB + ALND	2973 (31.3)	799 (24.6)	8935 (25.4)	
Median no. of LNs retrieved (IQR)	11 (5–16)	11 (5–17)	11 (5 - 17)	<0.001
Median no. of positive LNs (IQR)	2 (1–5)	2 (1–5)	2 (1 - 5)	<0.001

NAC, neoadjuvant chemotherapy; NET, neoadjuvant endocrine therapy; SLNB, sentinel lymph node biopsy; ALND, axillary lymph node dissection; IQR, interquartile range; LN, lymph node

^a^Percentages may not add up to 100 due to rounding or missing values

**Fig. 3:**
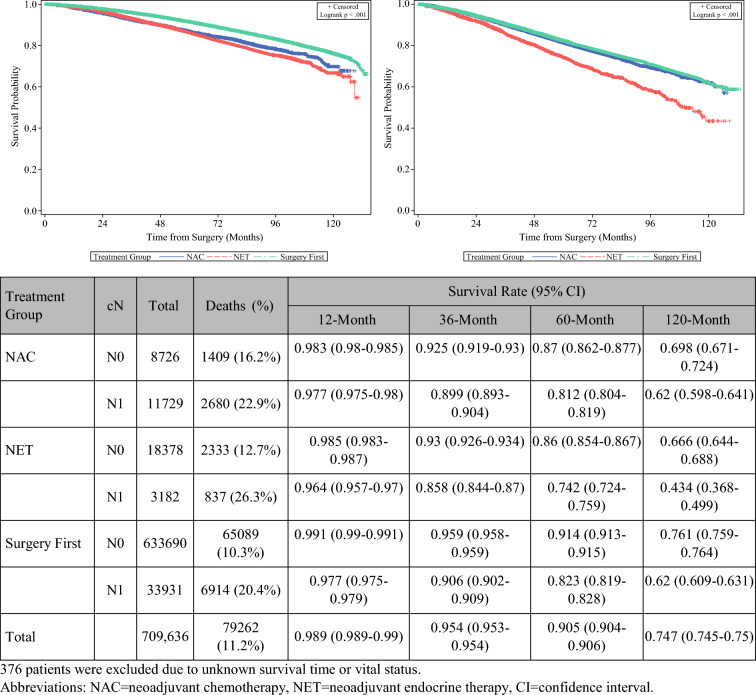
Unadjusted overall survival from time of surgery stratified by treatment group and clinical N category: cN0 (left), cN1 (right)

After 10 years of follow-up evaluation, unadjusted overall survival varied significantly by treatment group (*p* < 0.001, log-rank). Among the patients with cN0 disease, the highest survival rate was in the surgery-first group (76.1 %; 95 % CI, 75.9–76.4 %) compared with the NAC group (69.8 %; 95 % CI, 67.1–72.4 %) and the NET group (66.6 %; 95 % CI, 64.4–68.8 %). Among the patients with cN1 disease, the highest survival rate was in the surgery-first group (62.0 %; 95 % CI, 60.9–63.1 %) followed closely by the NAC group (62.0 %; 95 % CI, 59.8–64.1 %) and more distantly by the NET group (43.4 %; 95 % CI, 36.8–49.9 %) (Fig. 3).

**Fig. 4:**
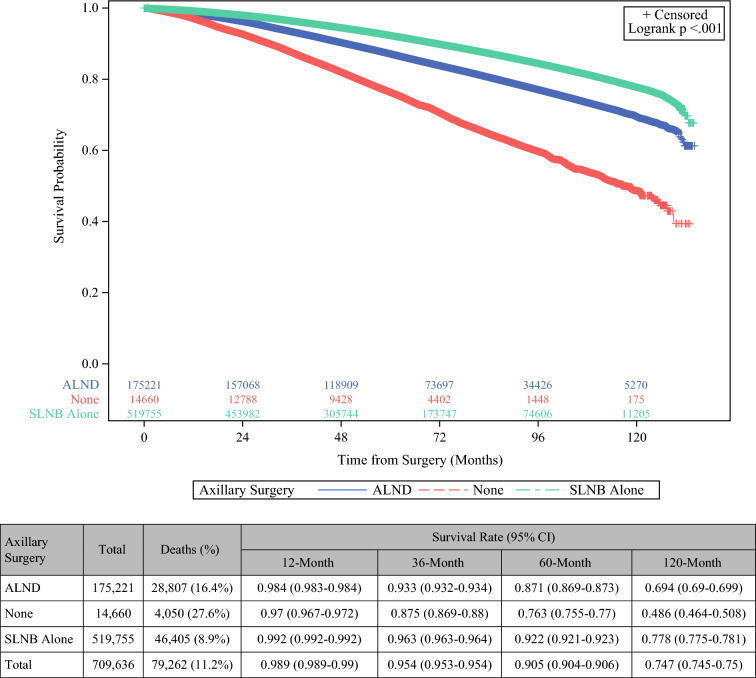
Unadjusted overall survival from time of surgery stratified by axillary surgery

Unadjusted overall survival also varied significantly by axillary surgery type (*p* < 0.001, log-rank). At 10 years, those who underwent SLNB had the highest survival rate (77.8 %; 95 % CI, 77.5–78.1 %) followed by those who underwent ALND (69.4 %; 95 % CI, 69.0–69.9 %) and finally by those who had no axillary surgery (48.6 %; 95 % CI, 46.4–50.8 %) (Fig. 4).

## Discussion

Neoadjuvant endocrine therapy can be an attractive alternative treatment for HR+/HER2– invasive breast cancer for select patients. Evidence exists to show that NET can provide similar efficacy in terms of tumor downstaging with decreased toxicity compared with NAC.^14,21,22^ This treatment strategy has been shown to improve rates of BCS for patients who would otherwise be ineligible.^23^ Additionally, we found that the median time from diagnosis to treatment was shortest in the NET group, likely due to ease of administration and less coordination required. This expedited treatment initiation can be advantageous for various reasons. Previous studies also have demonstrated that NET can be safely used as a bridge to surgery, such as during the COVID-19 pandemic.^24^ Despite these benefits, no clear guidelines on axillary management after NET currently exist, and this paucity of data was the target of our examination of recent data in the NCDB **(**Table 1).

We found that evidence-based efforts toward de-escalation of axillary surgery have resulted in lower rates of ALND over time regardless of neoadjuvant treatment type or upfront surgery. This is especially true at academic centers, which may be quicker to implement newer evidence-based findings and guidelines.^25^ For example, clinically node-positive disease historically required ALND, but currently, SLNB is recommended for select patients who can be clinically downstaged before surgery.^11,16^ Additionally, for postmenopausal women with up to three positive nodes, the incorporation of oncotype DX testing also may have contributed to selective de-escalation of systemic treatment with reduced emphasis on extensive axillary surgery as an extension of this treatment approach.^26^

We found that a much higher percentage of patients in the NAC group underwent ALND than in the NET and surgery-first groups. We hypothesized that this was due to what would be considered traditionally more aggressive disease (larger tumor size, increased nodal burden) treated with NAC followed by NET then surgery first (Table 2). Interestingly, even after adjustment for other meaningful variables, NAC still was associated with increased odds of treatment with ALND after reception of NAC compared with NET.

Until we have the results of Alliance 11202 and/or the TAXIS trial, patients with ypN+ disease are currently still advised to undergo completion ALND.^27–29^ However, we found that not all patients with residual nodal disease after SLNB went on to receive ALND. Among the clinically node-positive patients in this study, 19.6 % achieved nodal pCR after receiving NAC, compared to 8.3 % of those who received NET. Of those who did not achieve nodal pCR, only 70.5 % in the NAC group and 57.5 % in the NET group went on to completion ALND. Although findings have shown pCR to be a reasonable surrogate for overall survival in triple-negative and HER2+ breast cancer, it is a less appropriate surrogate marker for HR+/HER2– tumors, especially luminal A type tumors.^30,31^ Therefore, lack of pCR may not have as much influence in the decision to proceed with completion ALND, especially for patients treated with NET who receive only a small proportion of their systemic therapy upfront. Additionally, in the NET setting, tools such as the modified Preoperative Endocrine Prognostic Index (PEPI) score are increasingly used to guide treatment decisions given that PCR is a less reliable surrogate for long-term outcomes in HR+/HER2– breast cancer.^31–33^ This reasoning may explain such a discrepancy in the care observed in this study compared with the current guidelines.

Survival analysis indicated higher survival rates for patients undergoing surgery first for both cN0 and cN1 disease, likely related to patient selection and more readily operable tumors. Interestingly, the survival rates between those receiving NAC and those receiving NET were similar for cN0 disease, but for the individuals with cN1 disease, those receiving NAC had survival rates similar to those for the surgery-first patients, and those receiving NET had the worst overall survival. Although causation cannot be established from this retrospective analysis, this may reflect the selection of patients for NET who were older or had more comorbidities making them less likely to tolerate NAC.

Survival curves also differed significantly when stratified by axillary surgery, with the lowest survival rates for individuals not undergoing axillary surgery, likely because omission of axillary surgery was more common for older individuals and those with comorbidities. Survival was higher for the individuals undergoing SLNB than for those undergoing ALND, which is most likely representative of increased nodal disease in the latter.

The limitations to this study included its retrospective design with extraction of data from a large database representative of Commission on Cancer (CoC)-accredited intuitions, which may limit generalizability to other practices. The CoC accreditation focuses on meeting standards of multidisciplinary care, data collection, and quality improvement. As a result, the implementation of NET may be over-represented, and the implementation of axillary management after treatment may be different. The database also lacks certain granular details on the reason behind treatment selection, including patient preferences that may influence treatment decision-making. The NCDB also does not capture the use of specific agents, such as CDK4/6 inhibitors, and the number of individuals treated with such agents could not be accounted for or excluded. Finally, this observational study lacked the ability to draw causal inferences from the patterns elucidated in this analysis.

Collectively, however, the information in this report helps to inform the current state of the art of axillary management for NET and highlights areas for improvement in standardizing care guidelines. Despite the lower nodal PCR rates with NET, omission of completion ALND remains common in this group. This may be reflective of a tendency to treat this group similarly to the surgery-first group given that a much smaller proportion of systemic therapy is administered before surgery than with NAC. However, definitive conclusions as to the reason cannot be drawn, underscoring the need for evidence-based NET-specific guidelines for axillary management.

## Supplementary Information

Below is the link to the electronic supplementary material.

Supplementary file1 (DOCX 35 kb)

## Data Availability

All data used for this study are from the NCDB, which is freely available to CoC-accredited facilities upon request.
