# Supplementary material for: Contemporary Trends in Axillary Surgery for ER-Positive, HER2-Negative Breast Cancer Stratified by Neoadjuvant Endocrine Therapy, Neoadjuvant Chemotherapy, or Upfront Surgery
Source: Ann Surg Oncol. 2025 Sep 23;33(1):95–107. doi: 10.1245/s10434-025-18225-5 (PMC12619107; doi:10.1245/s10434-025-18225-5)
Supplement: Supplementary file 1 — Supplementary file1 (DOCX 35 kb) [file 10434_2025_18225_MOESM1_ESM.docx]

*Supplemental Figure(s):*

**Supplemental Table. Patient Characteristics by Axillary Surgery**

|  | **All Patients**  **(N=792,581)** | **ALND**  **(N=186,457)** | **SLNB**  **(N=590,827)** | **None**  **(N=15,297)** | **P-Value** |
| --- | --- | --- | --- | --- | --- |
| Age (Years) |  |  |  |  | <0.001 |
| 50-69 | 513,400 (64.8%) | 123,369 (66.2%) | 385,310 (65.2%) | 4,721 (30.9%) |  |
| 70+ | 279,181 (35.2%) | 63,088 (33.8%) | 205,517 (34.8%) | 10,576 (69.1%) |  |
| Median (IQR) | 66 (59 - 72) | 65 (58 - 72) | 66 (59 - 72) | 76 (67 - 84) |  |
| Race and Ethnicity |  |  |  |  | <0.001 |
| Hispanic | 37,054 (4.7%) | 10,104 (5.4%) | 26,336 (4.5%) | 614 (4%) |  |
| Non-Hispanic Asian | 23,516 (3%) | 5,133 (2.8%) | 17,971 (3%) | 412 (2.7%) |  |
| Non-Hispanic Black | 64,809 (8.2%) | 18,401 (9.9%) | 45,127 (7.6%) | 1,281 (8.4%) |  |
| Non-Hispanic White | 641,167 (80.9%) | 145,895 (78.2%) | 482,815 (81.7%) | 12,457 (81.4%) |  |
| Other | 7,585 (1%) | 1,907 (1%) | 5,551 (0.9%) | 127 (0.8%) |  |
| Unknown* | 18,450 (2.3%) | 5,017 (2.7%) | 13,027 (2.2%) | 406 (2.7%) |  |
| Income Level |  |  |  |  | <0.001 |
| <$48,000 | 231,829 (29.2%) | 60,898 (32.7%) | 166,639 (28.2%) | 4,292 (28.1%) |  |
| ≥$48,000 | 446,942 (56.4%) | 100,616 (54%) | 337,169 (57.1%) | 9,157 (59.9%) |  |
| Unknown* | 113,810 (14.4%) | 24,943 (13.4%) | 87,019 (14.7%) | 1,848 (12.1%) |  |
| Education Level |  |  |  |  | <0.001 |
| High School Graduation Rate ≤87% | 242,305 (30.6%) | 63,873 (34.3%) | 173,775 (29.4%) | 4,657 (30.4%) |  |
| High School Graduation Rate >87% | 436,702 (55.1%) | 97,703 (52.4%) | 330,203 (55.9%) | 8,796 (57.5%) |  |
| Unknown* | 113,574 (14.3%) | 24,881 (13.3%) | 86,849 (14.7%) | 1,844 (12.1%) |  |
| Insurance Type |  |  |  |  | <0.001 |
| Private | 337,186 (42.5%) | 79,976 (42.9%) | 253,500 (42.9%) | 3,710 (24.3%) |  |
| Government | 440,474 (55.6%) | 101,886 (54.6%) | 327,324 (55.4%) | 11,264 (73.6%) |  |
| None | 7,665 (1%) | 2,520 (1.4%) | 5,035 (0.9%) | 110 (0.7%) |  |
| Unknown* | 7,256 (0.9%) | 2,075 (1.1%) | 4,968 (0.8%) | 213 (1.4%) |  |
| Community Type |  |  |  |  | <0.001 |
| Metropolitan | 669,710 (84.5%) | 155,864 (83.6%) | 500,548 (84.7%) | 13,298 (86.9%) |  |
| Urban | 93,779 (11.8%) | 23,615 (12.7%) | 68,667 (11.6%) | 1,497 (9.8%) |  |
| Rural | 11,861 (1.5%) | 2,999 (1.6%) | 8,698 (1.5%) | 164 (1.1%) |  |
| Unknown* | 17,231 (2.2%) | 3,979 (2.1%) | 12,914 (2.2%) | 338 (2.2%) |  |
| Charlson/Deyo Comorbidity Score |  |  |  |  | <0.001 |
| 0 | 635,991 (80.2%) | 147,613 (79.2%) | 476,590 (80.7%) | 11,788 (77.1%) |  |
| 1 | 113,421 (14.3%) | 28,434 (15.2%) | 82,688 (14%) | 2,299 (15%) |  |
| 2+ | 43,169 (5.4%) | 10,410 (5.6%) | 31,549 (5.3%) | 1,210 (7.9%) |  |
| Year of Diagnosis |  |  |  |  | <0.001 |
| 2012 | 65,580 (8.3%) | 22,956 (12.3%) | 41,322 (7%) | 1,302 (8.5%) |  |
| 2013 | 72,036 (9.1%) | 23,608 (12.7%) | 46,993 (8%) | 1,435 (9.4%) |  |
| 2014 | 75,574 (9.5%) | 22,717 (12.2%) | 51,197 (8.7%) | 1,660 (10.9%) |  |
| 2015 | 79,747 (10.1%) | 22,139 (11.9%) | 55,704 (9.4%) | 1,904 (12.4%) |  |
| 2016 | 85,079 (10.7%) | 22,038 (11.8%) | 60,020 (10.2%) | 3,021 (19.7%) |  |
| 2017 | 87,765 (11.1%) | 21,136 (11.3%) | 63,585 (10.8%) | 3,044 (19.9%) |  |
| 2018 | 82,704 (10.4%) | 16,687 (8.9%) | 64,725 (11%) | 1,292 (8.4%) |  |
| 2019 | 86,614 (10.9%) | 13,266 (7.1%) | 72,776 (12.3%) | 572 (3.7%) |  |
| 2020 | 74,913 (9.5%) | 10,763 (5.8%) | 63,711 (10.8%) | 439 (2.9%) |  |
| 2021 | 82,569 (10.4%) | 11,147 (6%) | 70794 (12%) | 628 (4.1%) |  |
| Facility Type |  |  |  |  | <0.001 |
| Academic/Research | 224,541 (28.3%) | 52,037 (27.9%) | 168,197 (28.5%) | 4,307 (28.2%) |  |
| Community | 58,329 (7.4%) | 16,218 (8.7%) | 41,043 (6.9%) | 1,068 (7%) |  |
| Comprehensive Community | 334,681 (42.2%) | 79,914 (42.9%) | 248,210 (42%) | 6,557 (42.9%) |  |
| Integrated Network | 175,030 (22.1%) | 38,288 (20.5%) | 133,377 (22.6%) | 3,365 (22%) |  |
| Facility Location |  |  |  |  | <0.001 |
| Midwest | 206,664 (26.1%) | 47,678 (25.6%) | 155,863 (26.4%) | 3,123 (20.4%) |  |
| Northeast | 160,432 (20.2%) | 36,075 (19.3%) | 119,995 (20.3%) | 4,362 (28.5%) |  |
| South | 283,750 (35.8%) | 71,665 (38.4%) | 207,458 (35.1%) | 4,627 (30.2%) |  |
| West | 141,735 (17.9%) | 31,039 (16.6%) | 107,511 (18.2%) | 3,185 (20.8%) |  |
| Histology |  |  |  |  | <0.001 |
| Ductal | 581,668 (73.4%) | 132,226 (70.9%) | 438,535 (74.2%) | 10,907 (71.3%) |  |
| Lobular | 177,761 (22.4%) | 47,877 (25.7%) | 126,633 (21.4%) | 3,251 (21.3%) |  |
| Other | 33,152 (4.2%) | 6,354 (3.4%) | 25,659 (4.3%) | 1,139 (7.4%) |  |
| Clinical T Category |  |  |  |  | <0.001 |
| T1 | 611,721 (77.2%) | 114,992 (61.7%) | 484,447 (82%) | 12,282 (80.3%) |  |
| T2 | 157,584 (19.9%) | 57,439 (30.8%) | 97,577 (16.5%) | 2,568 (16.8%) |  |
| T3 | 18,436 (2.3%) | 10,764 (5.8%) | 7,430 (1.3%) | 242 (1.6%) |  |
| T4 | 4,840 (0.6%) | 3,262 (1.7%) | 1,373 (0.2%) | 205 (1.3%) |  |
| Clinical N Category |  |  |  |  | <0.001 |
| N0 | 739,621 (93.3%) | 144,887 (77.7%) | 579,741 (98.1%) | 14,993 (98%) |  |
| N1 | 52,960 (6.7%) | 41,570 (22.3%) | 11,086 (1.9%) | 304 (2%) |  |
| Pathologic T Category |  |  |  |  | <0.001 |
| T0 | 2,652 (0.3%) | 1,055 (0.6%) | 1,546 (0.3%) | 51 (0.3%) |  |
| T1 | 576,208 (72.7%) | 103,996 (55.8%) | 460,535 (77.9%) | 11,677 (76.3%) |  |
| T1IS | 1,416 (0.2%) | 368 (0.2%) | 939 (0.2%) | 109 (0.7%) |  |
| T2 | 183,995 (23.2%) | 64,104 (34.4%) | 116,932 (19.8%) | 2,959 (19.3%) |  |
| T3 | 24,203 (3.1%) | 14,137 (7.6%) | 9,780 (1.7%) | 286 (1.9%) |  |
| T4 | 4,107 (0.5%) | 2,797 (1.5%) | 1,095 (0.2%) | 215 (1.4%) |  |
| Pathologic N Category |  |  |  |  | <0.001 |
| N0 | 622,061 (78.5%) | 92,852 (49.8%) | 514,521 (87.1%) | 14,688 (96%) |  |
| N1 | 107,901 (13.6%) | 56,563 (30.3%) | 50,934 (8.6%) | 404 (2.6%) |  |
| N1Mic | 30,595 (3.9%) | 7,107 (3.8%) | 23,378 (4%) | 110 (0.7%) |  |
| N2 | 22,403 (2.8%) | 20,572 (11%) | 1,765 (0.3%) | 66 (0.4%) |  |
| N3 | 9,621 (1.2%) | 9,363 (5%) | 229 (0%) | 29 (0.2%) |  |
| Tumor Size (cm) – Median (IQR) | 1.4 (0.9 - 2.2) | 1.9 (1.2 - 3) | 1.3 (0.9 - 2) | 1.2 (0.8 - 2) | <0.001 |
| Number LNs Retrieved – Median (IQR) | 2 (1 - 4) | 7 (3 - 13) | 2 (1 - 3) | 0 (0 - 0) | <0.001 |
| Number Positive LNs – Median (IQR) | 0 (0 - 0) | 1 (0 - 2) | 0 (0 - 0) | 0 (0 - 0) | <0.001 |
| Grade |  |  |  |  | <0.001 |
| 1 | 247,278 (31.2%) | 43,845 (23.5%) | 198,125 (33.5%) | 5,308 (34.7%) |  |
| 2 | 411,649 (51.9%) | 99,133 (53.2%) | 304,952 (51.6%) | 7,564 (49.4%) |  |
| 3 | 113,378 (14.3%) | 37,055 (19.9%) | 74,528 (12.6%) | 1,795 (11.7%) |  |
| Unknown* | 20,276 (2.6%) | 6,424 (3.4%) | 13,222 (2.2%) | 630 (4.1%) |  |
| Lymphovascular Invasion |  |  |  |  | <0.001 |
| Absent | 603,664 (76.2%) | 117,774 (63.2%) | 473,754 (80.2%) | 12,136 (79.3%) |  |
| Present | 102,909 (13%) | 45,772 (24.5%) | 55,888 (9.5%) | 1,249 (8.2%) |  |
| Unknown* | 86,008 (10.9%) | 22,911 (12.3%) | 61,185 (10.4%) | 1,912 (12.5%) |  |
| PR Status |  |  |  |  | <0.001 |
| PR+ | 703,692 (88.8%) | 164,173 (88%) | 526,032 (89%) | 13,487 (88.2%) |  |
| PR- | 88,330 (11.1%) | 22,167 (11.9%) | 64,374 (10.9%) | 1,789 (11.7%) |  |
| Unknown* | 559 (0.1%) | 117 (0.1%) | 421 (0.1%) | 21 (0.1%) |  |
| Treatment Group |  |  |  |  | <0.001 |
| NAC | 20,704 (2.6%) | 13,053 (7%) | 7,418 (1.3%) | 233 (1.5%) |  |
| NET | 24,218 (3.1%) | 7,183 (3.9%) | 16,325 (2.8%) | 710 (4.6%) |  |
| Surgery First | 747,659 (94.3%) | 166,221 (89.1%) | 567,084 (96%) | 14,354 (93.8%) |  |
| Chemotherapy Type |  |  |  |  | <0.001 |
| None | 644,780 (81.4%) | 118,040 (63.3%) | 512,558 (86.8%) | 14,182 (92.7%) |  |
| Adjuvant | 127,097 (16%) | 55,364 (29.7%) | 70,851 (12%) | 882 (5.8%) |  |
| Neoadjuvant | 20,704 (2.6%) | 1,3053 (7%) | 7,418 (1.3%) | 233 (1.5%) |  |
| Endocrine Therapy Type |  |  |  |  | <0.001 |
| None | 97,206 (12.3%) | 23,036 (12.4%) | 69,812 (11.8%) | 4,358 (28.5%) |  |
| Adjuvant | 671,157 (84.7%) | 156,238 (83.8%) | 504,690 (85.4%) | 10,229 (66.9%) |  |
| Neoadjuvant | 24,218 (3.1%) | 7,183 (3.9%) | 16,325 (2.8%) | 710 (4.6%) |  |
| Treatment with Radiation |  |  |  |  | <0.001 |
| No | 279,906 (35.3%) | 72,956 (39.1%) | 1969,51 (33.3%) | 9,999 (65.4%) |  |
| Yes | 485,221 (61.2%) | 106,925 (57.3%) | 373,457 (63.2%) | 4,839 (31.6%) |  |
| Unknown* | 27,454 (3.5%) | 6,576 (3.5%) | 20,419 (3.5%) | 459 (3%) |  |
| Treatment with Immunotherapy |  |  |  |  | <0.001 |
| No | 785,878 (99.2%) | 184,008 (98.7%) | 586,681 (99.3%) | 15,189 (99.3%) |  |
| Yes | 6,259 (0.8%) | 2,282 (1.2%) | 3,883 (0.7%) | 94 (0.6%) |  |
| Unknown* | 444 (0.1%) | 167 (0.1%) | 263 (0%) | 14 (0.1%) |  |
| Surgery Type |  |  |  |  | <0.001 |
| Lumpectomy | 549,896 (69.4%) | 88,148 (47.3%) | 450,053 (76.2%) | 11,695 (76.5%) |  |
| Mastectomy | 242,685 (30.6%) | 98,309 (52.7%) | 140,774 (23.8%) | 3,602 (23.5%) |  |
| Time from Diagnosis to  Treatment (Months) – Median (IQR) | 1.18 (0.79 - 1.71) | 1.18 (0.79 - 1.78) | 1.18 (0.79 - 1.68) | 1.12 (0.69 - 1.64) | <0.001 |
| Time from Start of Endocrine Therapy to Definitive Surgery (Months) – Median (IQR)** | 1.81 (0.95 - 4.14) | 2.30 (1.05 - 5.23) | 1.64 (0.92 - 3.45) | 3.16 (1.09 - 6.25) | <0.001 |
| Follow-Up (Months) – Median (95% CI) | 62.5 (62.4 - 62.6) | 72.2 (71.9 - 72.4) | 59.5 (59.4 - 59.6) | 66.8 (66.1 - 67.5) | <0.001 |

Data reported as N (%) unless otherwise specified. Percentages may not add up to 100 due to rounding or missing values.

*Unknown values are not included in p-value estimation.

**Among NET patients only.

Abbreviations: ALND=axillary lymph node dissection, SLNB=sentinel lymph node biopsy, PR=progesterone receptor, NAC=neoadjuvant chemotherapy, NET=neoadjuvant endocrine therapy, IQR=interquartile range, CI=confidence interval.
